# Connected Health Services: Framework for an Impact Assessment

**DOI:** 10.2196/14005

**Published:** 2019-09-03

**Authors:** Ioanna Chouvarda, Christos Maramis, Kristina Livitckaia, Vladimir Trajkovik, Serhat Burmaoglu, Hrvoje Belani, Jan Kool, Roman Lewandowski, The ENJECT Working Group 1 Network

**Affiliations:** 1 Lab of Computing, Medical Informatics and Biomedical Imaging Technologies School of Medicine, Faculty of Health Sciences Aristotle University of Thessaloniki Thessaloniki Greece; 2 Faculty of Computer Science and Engineering, Saints Cyril and Methodius University Skopje North Macedonia; 3 Department of Health Management Faculty of Economics and Administrative Sciences Izmir Katip Celebi University Izmir Turkey; 4 IT Division, Ministry of Health Zagreb Croatia; 5 Rehabilitation Centre Valens Valens Switzerland; 6 Faculty of Management, University of Social Sciences Lodz Poland

**Keywords:** connected health, health impact assessment, framework, outcome assessment, enablers and barriers

## Abstract

**Background:**

Connected health (CH), as a new paradigm, manages individual and community health in a holistic manner by leveraging a variety of technologies and has the potential for the incorporation of telehealth and integrated care services, covering the whole spectrum of health-related services addressing healthy subjects and chronic patients. The reorganization of services around the person or citizen has been expected to bring high impact in the health care domain. There are a series of concerns (eg, contextual factors influencing the impact of care models, the cost savings associated with CH solutions, and the sustainability of the CH ecosystem) that should be better addressed for CH technologies to reach stakeholders more successfully. Overall, there is a need to effectively establish an understanding of the concepts of CH impact. As services based on CH technologies go beyond standard clinical interventions and assessments of medical devices or medical treatments, the need for standardization and for new ways of measurements and assessments emerges when studying CH impact.

**Objective:**

This study aimed to introduce the CH impact framework (CHIF) that serves as an approach to assess the impact of CH services.

**Methods:**

This study focused on the subset of CH comprising services that directly address patients and citizens on the management of disease or health and wellness. The CHIF was developed through a multistep procedure and various activities. These included, as initial steps, a literature review and workshop focusing on knowledge elicitation around CH concepts. Then followed the development of the initial version of the framework, refining of the framework with the experts as a result of the second workshop, and, finally, composition and deployment of a questionnaire for preliminary feedback from early-stage researchers in the relevant domains.

**Results:**

The framework contributes to a better understanding of what is CH impact and analyzes the factors toward achieving it. CHIF elaborates on how to assess impact in CH services. These aspects can contribute to an impact-aware design of CH services. It can also contribute to a comparison of CH services and further knowledge of the domain. The CHIF is based on 4 concepts, including CH system and service outline, CH system end users, CH outcomes, and factors toward achieving CH impact. The framework is visualized as an ontological model.

**Conclusions:**

The CHIF is an initial step toward identifying methodologies to objectively measure CH impact while recognizing its multiple dimensions and scales.

## Introduction

### Background Concepts

Presently, information and communication technologies, including a growing number of consumer and medical devices as well as patient services, have created new opportunities to improve the health and well-being of individuals and populations. Such improvements are expected to be successful through behavior change at a personal level, better health care coordination, and multilevel information sharing, gradually building the connected health (CH) landscape [1].

CH, as a new paradigm, manages individual and community health in a connected and holistic manner by leveraging a variety of technologies [2,3]. CH is a promising vehicle for the incorporation of telehealth and integrated care services, covering the whole spectrum of health-related services from the ones directing the *healthy subject* (as a citizen who seeks health service support or a wellness service consumer) to those addressing the *chronic patient* as an integrated (tele) care service beneficiary. The evolution of the CH ecosystem and related concepts (eg, telemedicine) has been discussed from a bibliometric viewpoint in a study by Burmaoglu [4].

The reorganization of services around the person or citizen, with person-centered care being a promising area [5], is expected to bring an important impact in the health domain. This will require addressing a series of concerns in an effective manner to more successfully reach stakeholders: contextual factors influencing the impact of care models, the cost savings associated with CH solutions, and the sustainability of the CH ecosystem. Overall, there is a need to shed light on the concepts of CH impact.

### The Aim

This paper aimed to introduce the CH impact framework (CHIF) that serves in the assessment of CH services’ impact. CHIF was born from the European Network for the Joint Evaluation of Connected Health Technologies (ENJECT), a network actively involved in the evaluation of CH technologies, funded by European Cooperation in Science and Technology (COST) Action [6]. Within the complete spectrum of CH, this study focused on the subset of CH comprising *services that directly address patients and citizens* at large on the *management of disease or health and wellness*. These CH patient services are heavily dependent on new technologies. Nevertheless, CH services are not considered detached from established health information technology (either secondary care medical technology or technology primarily oriented to the health care professional [HCP], such as ePrescription) and the respective health care services.

The reason for this study focusing on CH patient services, on the verge of consumer informatics, was because this is a new, highly promising area that is unmapped and in a gray zone concerning health care, meaning that there are no explicit care models incorporating CH services, health policies, or guidelines or standard ways of assessing these services.

### Related Work and Rationale

When considering health impact assessment (HIA) of a policy, program, or project, its potential effects on the health of a population and the distribution of those effects are evaluated [7,8] so as to produce (1) recommendations supporting decision makers and other stakeholders in making choices about alternatives and (2) improvements to avoid risks, prevent disease or injury, and actively promote health. The impact of CH technologies and services needs to be well defined [9], providing relevant evidence linking to and extending the HIA procedures.

On a broader scale, a relevant study regarding the assessment of integrated care services and scaling up of integrated care in European regions has been conducted by the European Innovation Partnership on Active and Healthy Ageing B3 [10] group (the Action Group on integrated care). The topics addressed included the following: (1) assessment of the health care system’s capacity to adopt integrated approaches to deal with challenges of aging, (2) assessment of the uptake of a particular good practice by a health care system, (3) identification of maturity characteristics necessary for adoption and scale-up of good practice, and (4) understanding the context and conditions in adopting and transferring practices among regions. The Maturity Model of B3 group was developed [11] as a tool to assess maturity along 12 dimensions reflecting the various aspects that need to be managed to deliver integrated care.

In addition, with regard to the assessment of telemedicine applications and services, the Model for Assessment of Telemedicine (MAST) tool was developed [12] to describe the effectiveness of telemedicine applications and their contribution to the quality of care. MAST summarizes and evaluates information about the medical, social, economic, and ethical issues related to the use of telemedicine, considering 7 assessment domains (ie, health problem, safety, clinical effectiveness, patient perspectives, financial aspects, organizational aspects, and sociocultural, ethical, and legal aspects). A framework for the emerging area of behavioral interventions was recently proposed [13], yet not elaborating on impact. Methodological aspects for CH evaluation were introduced by O’Leary [14] and Carroll [15], although not uniquely focusing on impact. Consumer health informatics assessment is discussed in the study by Gibbons et al [16]; this includes users, barriers at the system and individual level, implementation of applications (ie, function and process), and outcomes at different levels and directions (intermediate, health care processes, and clinical, economic, and relationship-centered).

There are not many papers that specifically refer to CH and its impact or to the use of specific frameworks for the impact of CH. In the CH review of Colorafi [17], the theoretical construct of a study by Ryan and Sawin [18] for self-management is adopted. It applies to both chronic conditions and health promotion and considers work, context, process, and proximal and distal outcomes. More specifically, according to this framework, self-management takes place in the context of (1) risk and protective factors specific to the condition, (2) a particular physical and social environment (eg, health care access, culture, and transportation), and (3) a set of individual and family factors (eg, literacy and family structure and capacity to self-manage). Self-management is a process involving individuals and families that includes (1) knowledge, facts, and beliefs (eg, self-efficacy), (2) self-regulation skills and abilities (eg, goal setting, decision making, and emotional control), and (3) social facilitation, including influence, support, and collaboration, to achieve positive health-related outcomes. Interventions to the person and family consider both process and context. The proximal or short-term outcomes lead to the achievement of distal outcomes. Thus, a temporal causal relation is introduced. Proximal outcomes mainly include individual and family self-management behaviors, such as engagement in activities and recommendations of treatment, symptom management, and adherence to recommended pharmacological therapies. Secondly, engagement in health-related behaviors may positively impact the cost of health care services in the short term. The distal outcomes are threefold: (1) health status as an indicator of the disease trajectory (indicating prevention, attenuation, stabilization, and worsening of the condition), (2) quality of life and perceived well-being, and (3) direct and indirect costs.

In the same vein, as services based on CH technologies go beyond standard clinical interventions and assessments of medical devices or medical treatments, the need for standardization and for new ways of measurements emerge when studying CH impact in depth. As mentioned in the study by Colorafi [17], “we likely need more sophisticated study designs if we are to adequately assess which element of a comprehensive program is affecting the outcome, asking how exactly do the ‘ *interventions impact the psychosocial aspects of the lives of people with diabetes?* ’.” Therefore, this area needs further research and disambiguation, especially, with regard to outcomes and impact. Although the abovementioned efforts are relevant to the concept of CH and offer valuable insights, CH services constitute more complex constructs that are not compartmentalized and assessed in the same manner as pharmacological trials.

The emerging CH technologies impose the definition of a CHIF. The CHIF was created in the process of exploring concepts around *what CH impact is* and *how it can be described*, *assessed*, and *achieved*. Specifically, CHIF is based on the inputs from the 2 workshops conducted in the scope of ENJECT within the last 2 years. In the following sections, the steps taken for deriving CHIF are presented, and the framework itself is described in detail along with a preliminary assessment tool based on CHIF. The paper also discusses challenges and future steps.

## Methods

**Figure 1:**
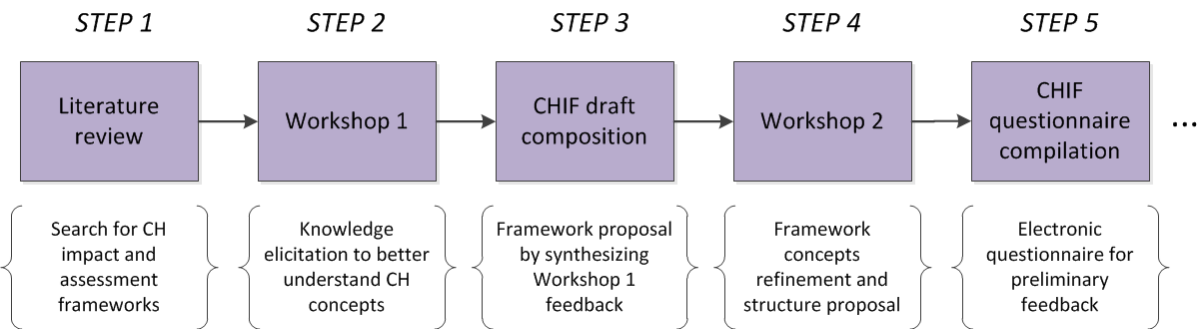
Methodological steps for the derivation of connected health (CH) impact framework.

The formulation of the CHIF framework took place in a multistep process, as delineated below, and it is visually outlined in Figure 1:

- Step 1: A literature review was conducted on the topics of CH impact and assessment frameworks. This step helped identify the main concepts and issues discussed in the domain and helped us further shape our research.
- Step 2: A workshop for further knowledge and insight elicitation toward better understanding the concepts around CH impact was conducted (workshop 1). The methodology employed was based on the structured group feedback approach [19].
- Step 3: Following workshop 1, knowledge elicitation took place, which resulted in a proposal for a CHIF based on a synthesis of inputs.
- Step 4: In workshop 2, the first CHIF proposal was presented and discussed among the participants, and this was the basis for the further effort to organize and propose the framework for reporting impact, including discussion and refinement of the previously established concepts and framework structure. The result was the consolidation of the CHIF structure. In addition, different visual representations of CHIF were suggested, for example, the ontological model.
- Step 5: CHIF was implemented in an electronic questionnaire for preliminary feedback.

More details about the workshops can be found in the Multimedia Appendix 1.

## Results

### Overview of the Connected Health Impact Framework

The CHIF serves in the assessment of CH services impact. It aims to contribute to (1) a better understanding of what is CH impact, (2) exploring how to achieve impact and thus support in better designing of CH services, and (3) methods to measure and assess impact, which can also help compare the impact of CH services and gather further knowledge. To meet these aims, 4 axes are considered: (1) CH system or service outline—of note, both the concepts of *system* and *service* are mentioned, as the focus is sometimes on the developed application and sometimes on the provided service, which adds a broader scope, (2) CH system end users and their profile, including the profile of primary users that the system targets and secondary users, (3) CH outcomes and measures of impact at different levels, and (4) factors toward achieving CH impact, including barriers and enablers, and a clear value proposition.

**Figure 2:**
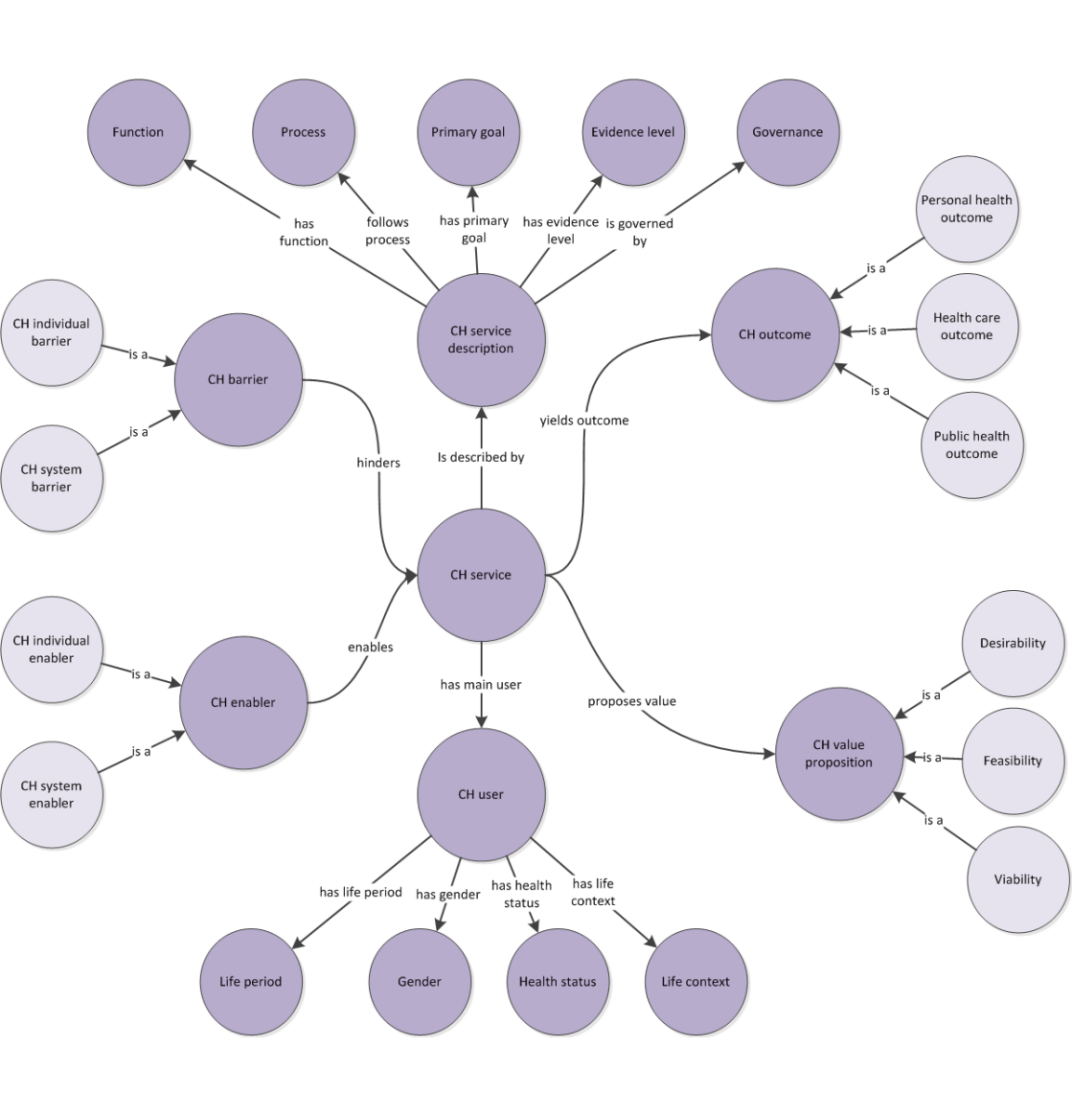
The ontological model of connected health impact framework; the framework is organized as a tree whose root is the concept of connected health (CH) system and service. Nodes beyond the third tree level are not depicted here, to reduce the complexity of visualization. Note that the arrows tagged as is a denote a subsumption relationship (ie, lighter colored nodes are subclasses of darker colored nodes).

The framework is visualized as an ontological model (see Figure 2). CHIF is organized as a tree with the concept of CH service at its root. Nodes beyond the third tree level are not depicted in the figure to reduce the complexity of visualization; however, all the nodes are presented in the following sections.

### Connected Health Impart Framework Axes

The following subsections refer to the description of the CHIF.

#### Connected Health System and Service Description

As a prerequisite, CH services need a basic level of functionality description.

We propose 5 elements, helping to describe a CH service through its *function, process*, *primary goal*, *evidence level*, and *control*. The *function* element reveals the functionality behind the service, such as assessment or monitoring, knowledge building, disease or condition management, or lifestyle management. The *process* element is responsible for describing how the function is implemented (eg, receiving a measurement from the user and returning automated feedback to the user). This element can include specific components supporting user personalization, such as social interactions and other. The *primary goal* reflects the health-related intent for optimization (eg, daily activity through the number of steps a day and night sleep duration). The *evidence level* describes the validation and evaluation of the service, including technical validation, clinical testing, and user experience. The last proposed element, *control*, refers to the governance of the service on a higher level. The control may belong to the patient or consumer, health care representative, social services, or payers, depending also on the CH services’ funding (private or public insurance).

These CH functionality elements can be directly or indirectly linked to impact and further support a better understanding of the service as well as compare services.

#### Connected Health Users

When addressing the personal CH outcomes coming from a specific technology or service, one has to explicitly specify the offered functionality and aim, as well as the users it addresses or applies to. Particularly, with regard to services and interventions, the targeted users should be well described. It is necessary to note that CH has many *contextual factors* influencing its adoption that should be reflected. These include geographical, social, demographics, human factors, educational, regulation, interoperability, and big data contexts of the specific CH systems within particular deployments. The contextual factors influence the clinical trials concerning the CH. Similarly, CH systems might vary significantly in different geographical regions, because of, for example, different environmental influences (eg, a training or coaching application should provide a different sports suggestion for the desert region). The same argument is valid for different demographics and socioeconomic contexts. The acceptance of services is often determined by human factors, such as the engagement, education level, and—especially—digital literacy of the end users. These contextual factors are essential to understanding that, for example, *the CH system X is efficient for female elder users with dementia in rural areas*. This fact is crucial when designing the system and reporting outcomes [20], especially if aiming at personalized interventions and avoiding *one-size-fits-all* ones.

We propose an initial approach where the *primary user* (ie, patient or citizen) is described with 4 elements: Phase of *life (young, working, retired,* or *dependent)*, *gender*, *health status (healthy, chronic patient, comorbid, acute disease,* or *disabled)*, and *life context*. The concept *young* includes childhood, adolescence, and transition to young adulthood. The last element comprises a list of factors that help describe the life circumstances of the user, including the location of living (eg, rural area), social activity, financial status, and others.

When not targeting consumer apps but health services, other involved users have to be identified, in addition to the direct beneficiary (ie, patient or citizen). The *secondary users* could be HCPs, the state, and policymakers, as well as businesses. CH may have an impact on all the user groups.

#### Connected Health Outcomes

**Figure 3:**
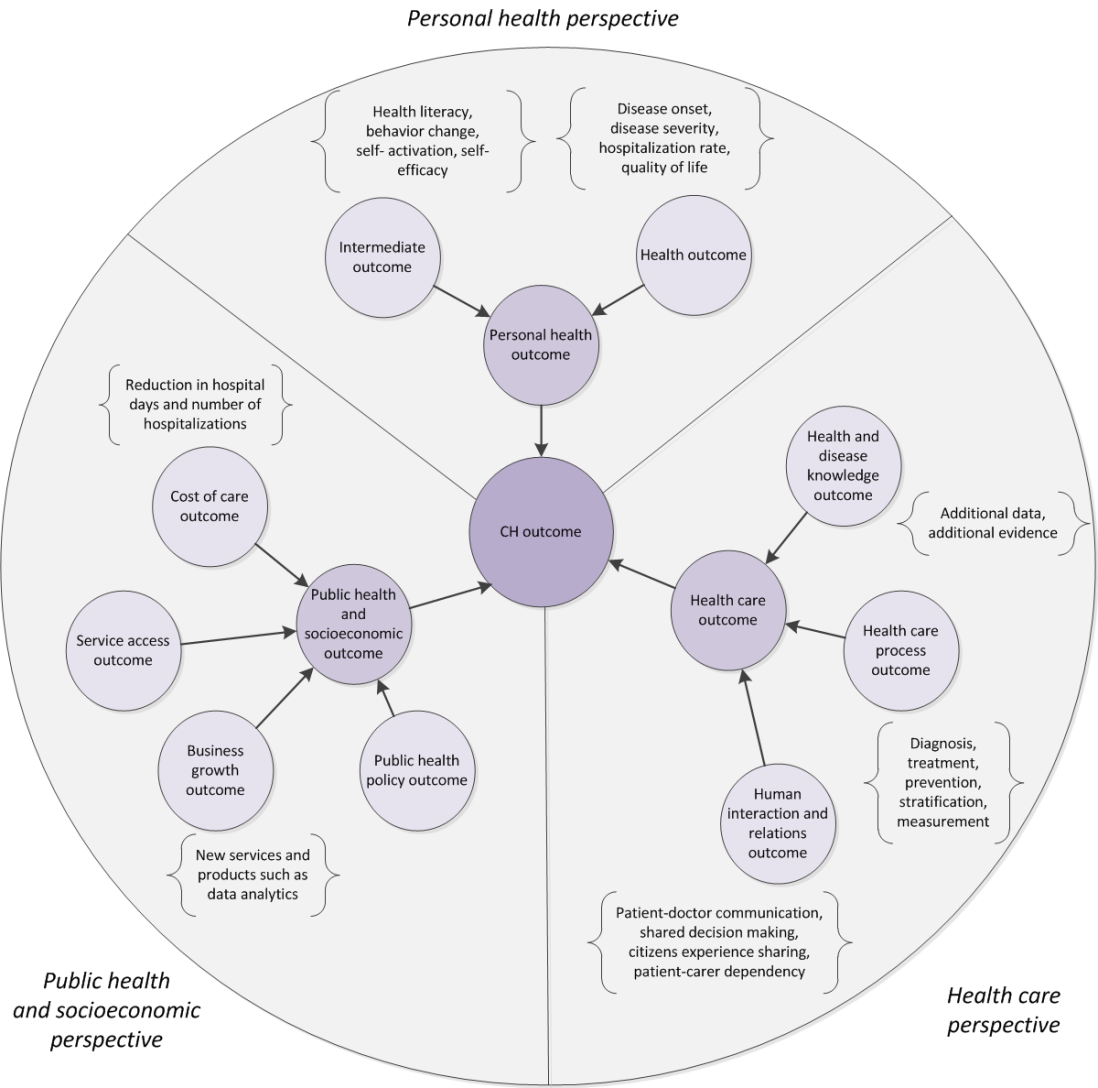
Perspectives on health-related outcomes that are associated with the impact of connected health (CH) services. All arrows denote is a relationships.

The health-related outcome of a CH service can be viewed from 3 different perspectives: (1) the personal perspective, (2) the health care process–related perspective, or (3) the wider socioeconomic or public health perspective (see Figure 3). These outcomes belonging to the 3 different perspectives are potentially intertwined.

##### Personal Outcomes

First, CH can affect the patient’s or citizen’s empowerment and engagement, as well as compliance with treatment [21,22] or other health behavior. These outcomes are expected to lead to the promotion of a healthy lifestyle, with further positive care and social consequences, improved health, and a better quality of life. In this regard, personal health outcomes are divided into the following categories: (1) intermediate outcomes (health literacy, behavior change, self-activation, and self-efficacy) [23] and (2) health outcomes (disease onset, disease deterioration, hospitalization rate, and quality of life), where the former is considered as potential mediators of the latter.

The introduction of CH tools may bring improved self-efficacy, understood here as a person’s ability to implement situation-specific behaviors toward attaining established goals, expectations, or designated types of outcomes [24]. Individuals knowing more about their health status may better cope with their health-related problems by themselves. Improved knowledge and understanding about health indicators, achieved through CH, while a person suffers from health problems can also reduce uncertainty in illness.

The timescales are expected to differ depending on the 2 types of the outcome, and many pilot studies decide to report on either but not both. However, reporting on both effects would help better understand the mechanisms of outcome formation and its further impact on the personal level.

A significant challenge is how to best measure these outcomes in a consistent manner, including both subjective or qualitative parts that are mostly measured with questionnaires and objective parts that are quantitatively measured through the use of various devices (eg, number of steps on a pedometer or heart rate on a smartwatch). Another challenge is when to measure the outcomes and, more importantly, how to express their temporal nature. Importantly, personal health outcomes are also linked to the care process outcomes (eg, improved access and accessibility to health care services), especially when combined with health literacy [25].

##### Health Care Process Outcomes

The utilization of CH relates to better patient safety, decreased duration of diagnostic processes (eg, early diagnoses), and better disease management (eg, identification of the risk of deterioration and primary and secondary prevention of disease) [26,27]. In the scope of CH, it is also expected to offer better access to the data, which can be used to improve understanding of the disease (especially in the case of chronic diseases) and provide evidence for health policy makers and other involved stakeholders. CH technologies offer great opportunities for a unified collection of patient-reported outcomes, which can affect the health care process [28].

The introduction of CH services impacts the models of care by enabling novel pathways for health monitoring, which include new interaction models supporting the involvement and empowerment of all stakeholders. These perspectives outline the need for novel clinical health care and social care guidelines, which can influence long-term health strategy design by promoting the economic efficiency of these services.

Overall, the directions identified as regards the care-related impacts of CH can be organized in 3 axes: (1) health care process (diagnosis, treatment, prevention, stratification, and measurement of outcome) [29], (2) human interaction and relations (patient-doctor communication and shared decisions, information and experience sharing, and patient-carer dependency), and (3) new health and disease knowledge (more data and evidence)

##### Public Health and Socioeconomic Outcomes

There are also horizontal aspects in CH outcomes, which affect multiple stakeholders and levels of health, and thus can be considered both as drivers and outcomes of the CH. These mainly include the facilitation of communication and information flow between health stakeholders and the improvement of health data analytics and management.

A characteristic example is the MyData Nordic Model [30]. This is an infrastructure for human-centered personal data management and processing, aiming to provide individuals with the practical means to access, obtain, and use datasets containing their personal information (ie, medical records, financial information, and data derived from various Web-based services). This approach introduces interesting dynamics at the societal and business level. Another example that relates CH data to public health policies is the BigO program, a European research project that analyzes daily living behavioral patterns of the youth to propose optimal physical activity–, diet-, and nutrition-related policies [31].

Cost reduction as an outcome can be expected at different levels, from the personal to the level of public health. There are studies on the cost-effectiveness of various telemedicine services. The main CH horizontal socioeconomic effects include (1) reduction of cost of care (eg, reduction in hospital days and number of hospitalizations), (2) improved and cost-efficient access to services, (3) improved public health policy, and (4) industrial activity and business growth, related to new services and products (eg, analytics services).

#### Factors Toward Achieving Connected Health Impact

##### Connected Health Value Proposition

To achieve scalability and impact, CH value proposition must be clearly articulated. In this respect, it is important to elaborate on what critical information regarding the CH applications is required for understanding the value proposition pertinent to each of the different stakeholders (eg, consumers and patients, their families, clinicians, developers, and payers). This is a clear statement of how the proposed solution relates to some improvement for the user, what specific benefits it brings, and how it differentiates from others. Although the value proposition is a consumer informatics concept, rather than a health care one, this concept may help crystallize the virtues of the CH application and its adoption.

As suggested in a McKinsey report [32], the 3 main properties that generally describe the value proposition of a CH solution are as follows: (1) *desirability* for all involved users (custom-centered and easy to use), (2) *feasibility* both technical and organizational, and (3) *viability* and sustainability (eg, via a supporting ecosystem, involving smart elements, and involving integration and collaboration of stakeholders).

To support these properties, the new solutions should be designed following a user-centered approach to (1) *respect the activities* a potential user has to perform, (2) *meet the expectations* (eg, comfort of use and easy to learn how to use), and (3) *minimize the fears* associated with the solution (eg, fears related to the new technology and fear of high costs). A CH service or product should be proposed based on the abovementioned elements.

##### Barriers and Enablers of Connected Health Impact

**Figure 4:**
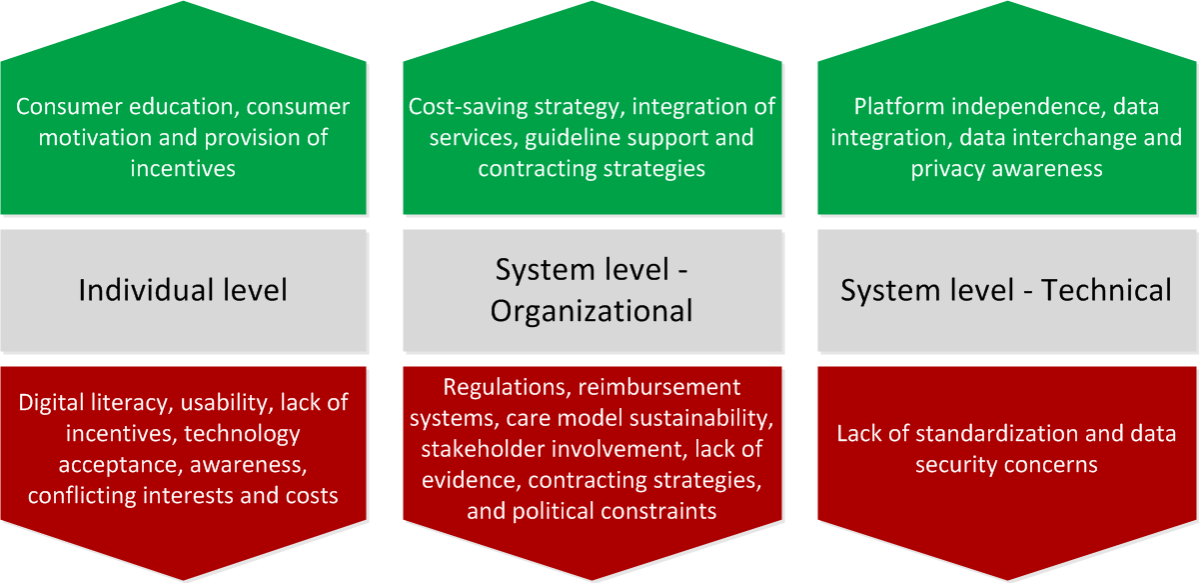
Classification of enablers (upward arrows) and barriers (downward arrows) of connected health impact as identified by the connected health impact framework; Individual (left) and system-level (right) enablers and barriers have been identified.

Figure 4 provides a visual outline in the CH impact enablers and barriers that have been identified by CHIF.

When designing and later evaluating a CH service and system or an application, it is necessary to recognize and report barriers that users (clinicians, developers, consumers, their families, caregivers, and policy makers) encounter and that can potentially limit the implementation or utilization of the CH solution. Considering *the user-CH system dipole*, we propose to classify the barriers, as *individual level* ones related to internal user features and abilities (eg, literacy gaps) and barriers external to the user and attributed at the *system level.* The latter might be *technical* (eg, design problems that limit usability) or *organizational* (eg, regulations). The individual level barriers include *digital literacy*, *usability*, *lack of incentives*, *technology acceptance*, *awareness*, *conflicting interests*, and *costs*. A series of system-level barriers related to an organization have been identified, including obstacles in *regulations*, *reimbursement systems*, *care* *model sustainability*, *stakeholder involvement*, *lack of evidence*, *contracting strategies*, and *political constraints.* Significant technical barriers at the system level include, among others, lack of standardization and data security concerns.

The enablers that a CH system employs toward achieving impact can be reported at the same levels as the barriers, that is, *individual* and *system* (categorized as *organizational* or *technical*) level, and span beyond merely overcoming technical challenges to address the problems identified at these levels and contribute to their solution. At the individual level, it is essential to *educate and motivate* consumers, potentially through incentives, for example, via offering a clear user-perceived health-related benefit.

At the system level, both organizational and technical issues need to be addressed. From the organizational side, issues within the health care organization (eg, *cost-saving strategy, integration of services,* and *guideline support*) need to be identified, and facilitating factors should be clearly defined. A vital organizational enabler is *contracting strategies*. In the developed countries, health care is delivered by the state, and most of the medical services are bought by a paying organization (payer) on behalf of patients or consumers as a third party of the transaction. This method shows that end users are not a party in the contract and are not directly interested in cost savings. Therefore, contracting strategies, as a way in which medical services are reimbursed, have to incentivize providers to implement innovative CH solutions. Payers already use many types of contracts that promote a different kind of provider behavior, for example, capitation, pay for performance, and case-mix contracts (diagnosis-related group, health regulation division, and shared saving models). On the technical side, best practices such as platform independence of applications, data integration and interchange, privacy awareness [33] are straightforward CH enablers.

Although proactively working to leverage enablers and overcome barriers, based on previous experience, the barriers and enablers are not in a static relation to the CH system. Besides, their relationship is not readily observable and quantified, that is, the power of the association, the extent to which they affect each other, and the outcome at a user level and beyond.

### Connected Health Impact Framework Preliminary Evaluation

To examine how understandable and usable the CH concepts and terms of the proposed framework and CHIF in general are, we developed an *electronic questionnaire assessment*, which followed a semistructured form [34]. The questionnaire, which also encouraged insights entered in free text, was introduced to early-stage researchers that work in the domain of CH in multiple disciplines (information technology, business, and health), in the scope of the ENJECT summer school (London, September 2017). A screen capture of the electronic questionnaire is provided in Multimedia Appendix 2. The questions included in the questionnaire are provided in Multimedia Appendix 3. The actual questionnaire was eventually completed anonymously by 5 volunteers from this group. The summarized answers are presented in Multimedia Appendix 4.

Although we received a limited number of responses, it helped in observing how the CHIF concepts were perceived. Analyzing the answers of the assessment, we noticed a pattern—most of the participants tended to fill in specific parts of the questionnaire, whereas other parts were consistently left without an answer. We assume that the answered questions represent the concepts of the CHIF that are understood and accepted, and the questions that remained unanswered suggest the concepts and terms of the framework that were not clear enough to understand. The answered questions were associated with the following concepts: CH User, CH outcomes toward impact, and partially the means to achieve impact. The parts presenting gaps in the answers included the primary goal of the framework, information on time scales of the different effects, industrial and business growth dimensions, and barriers and enablers beyond the patient or consumer user (system and secondary users). The value proposition section was left unanswered. Open questions were mostly unanswered.

Although preliminary, this limited assessment indicates the directions for improving the framework (especially the fuzzy areas) and shows the need for a better understanding and contemplating around the concept of CH services and their impact, beyond a solely technological perspective.

## Discussion

### Principal Findings

This paper provides an overview of CH impact concepts and proposes the CHIF for the consistent description and assessment of CH services’ impact in its different dimensions. Although this framework may benefit from further refinement, it is an attempt for setting the basis for complete and consistent reporting of this rather vague area, and it is expected to contribute in better evidence building and better designing of CH services.

A series of steps is foreseen that will lead to the CHIF deployment and use. The development of CHIF ontology is necessary for knowledge standardization and interoperability that lies in the very heart of CH. Following the example of mobile health (mHealth)–reporting guidelines [35], CHIF has the potential to evolve as a tool for reporting CH impact, either as a checklist or as an eQuestionnaire. This will also be useful for comparing interventions. In this regard, it can be part of a broader CH framework and form the basis for a digital registry of CH interventions to be further studied and compared.

An important step before that is to proceed with a thorough evaluation of CHIF-based tools in terms of clarity, completeness, and redundancy. The existing questionnaire will be the basis for that. A future evaluation will include compare between free text and structured entry of CH impact information and a post reporting questionnaire for user experience.

The following subsections discuss different aspects regarding CH impact that present challenges and could benefit from further investigation.

### The Multiple Dimensions and Scales of Connected Health Impact

We see CH impact as a multilevel concept, where potentially some CH outcomes at one level can influence those at other levels. The above interaction indicates that outcomes can also include causative relations. In essence, this requires approaching the concept of impact in a different manner: moving beyond the static clinical aggregate key performance indicators, toward linking outcomes in a more dynamic way (ie, personal, health care, and socioeconomic as well as horizontal aspects of the outcomes). Such approaches can be envisioned within a big data framework that can potentially reshape health policies. Among others, it could help to better elaborate on how each type of health outcome is linked with potential care benefit and cost reduction.

The concepts and directions stated above can set the basis for a CH taxonomy. The taxonomy in its turn can support consistent reporting, evidence building, and systematic reviewing purposes. Similar approaches have been used in consumer health [36], integrated care, and behavioral informatics applications [13].

One of the most crucial and challenging issues is the multiscale character of the CH impact. The impact can be at different structural (eg, micro-, meso-, or macroscale) and temporal scales. For example, a CH system can provide a more efficient cost-benefit ratio in the midterm to long term because of the initial increase of the cost of service and even more increased benefits and savings in the long term, owing to the abundance of information and knowledge produced by CH services.

### Connected Health Beyond Consumer Health Applications

CH extends beyond primary and secondary health care settings to the whole daily life, and therefore, inevitably uses technology that is beyond medical devices as established in clinical care. The *person-centered care* approach is well-suited to utilize consumer health technologies [37]. The role of consumer health electronics and systems in daily life has been recently recognized and appreciated. As described in the study by Gibbons et al [16], *consumer health informatics applications or tools* were defined as any electronic tool, technology, or system, which is in accordance with the following: (1) primarily designed to interact with health information users or consumers (ie, anyone who seeks or uses health care information for nonprofessional work), (2) interacts directly with the consumer who provides personal health information to the system and receives personalized health information from the tool, application, or system, and (3) the data, information, recommendations, or other benefits provided to the consumer may be used in coordination with an HCP but is not dependent on an HCP. In this regard, patients (individuals who have entered in the health care process) are distinguished from citizens and consumers.

By repurposing or extending their initial aims, such tools can be used and have already been used (eg, smartwatches and activity trackers) for (1) disease management to facilitate knowing, tracking, or understanding clinical parameters, (2) monitoring and understanding daily living observations (quantified-self perspective), (3) lifestyle management assistance (eg, calendar, reminder), (4) prevention and health promotion, (5) self-care, and (6) assisted care and caregiving. When considering the quantified self, socialization, or patient–health professional relationship domain, few published studies have investigated the determinants of the efficacy of these smart connected devices and their impact on individual behaviors and professional health practices [38].

A valid point for disambiguation is whether *CH basically is driven by consumer health electronics and applications*, a point extensively discussed in ENJECT workshops. The answers and views seem contradictory. From one side, it is believed that CH impact is mainly driven by consumer electronics in daily life, in other words, CH impact heavily relies on consumer health informatics. The reasoning behind this is that information and communication technologies and consumer electronics indeed influence and facilitate different aspects of everyday life and societal needs, including health. The culture of permanent self-monitoring (quantified self) is a typical case of this transformational power.

There is also an opinion about consumer electronics as a partial or moderate contributor to the broader impact of CH. The main arguments for the partial contribution are the lack of de-facto integration of consumer electronics data with medical data and also the lack of actionability at the medical level (professional interpretation of the data).

### Connected Health Impact Beyond Electronic and Mobile Health

Electronic health (eHealth) has been the generic platform for organizing and delivering digital health content and electronic and remote care services, whereas mHealth contributed to the wealth of mobile services focusing on the patient, the elderly, and the continuity of chronic care. The added value that CH can bring to the previous efforts in the eHealth and mHealth domain seems to span across 3 axes: (1) data and service integration and interaction, (2) validation of health-related services, and (3) overall health.

#### Data and Service Integration and Interaction

Traditionally, the technological framework for standardization and interoperability has been built within eHealth (eg, the data exchange standard health level 7 (HL7) [39]. However, from a functional perspective, the integration and interaction between personal and clinical information in a continuum, instead of overlapping eHealth, mHealth, and telemedicine, is a central point in CH. To a certain extent, this interaction can be regarded as a transfer of evidence from self-management data to the clinical treatment of patients and vice versa. Although this can now be technically leveraged by HL7 FHIR (Fast Health Care Interoperability Resources) and similar technology and standards [40], neither the organizational capability that is required on the health care side nor the scientific evidence on the use of such resources is entirely evident.

#### Validation of Health-Related Services

This can be regarded as a secondary outcome of data and service integration and interaction, supporting CH evidence formation.

#### Health

The CH services have the potential to contribute to the improvement in the diagnostic process (eg, shorter time to diagnosis), wellness, and evidence of self-management. Τelemedicine services for the elderly and patients with chronic diseases and those targeting accessibility to health care services (eg, people with disabilities or rural area residents) have been recognized and adopted to some extent. Other aspects, including patient and consumer empowerment, treatment adherence, prevention of behaviors contributing to health-related risks, and health literacy, are candidates future research and development targets toward achieving impact.

Overall, CH impact beyond eHealth and mHealth should focus on integration and access to a wealth of information and services. Therefore, there is a need for the explicit descriptions of services and data that will be linked and integrated, from both, technical and organizational perspective.

### Which Future Research Activities Can Facilitate Connected Health Impact?

CH is a new promising direction for improved health and well-being services [2]. Therefore, further research and investigations should concentrate on how CH can be interwoven into other important initiatives leading to cost containment and improvement of care.

Person-centered care and health promotion are both vital fields where CH tools are potentially able to prove their usefulness. CH in person-centered health care systems can support patients or consumers to cope with the health and well-being problems using their own resources, and as needed, help make informed decisions on when to invite others, including professionals, to act on their behalf. In this approach, well-designed CH tools may be able to prolong the period when patients and consumers would be capable to successfully manage their health and care according to their lifestyle, preferences, and goals. Patient-centered design and patients’ and consumers’ data analytics are the essential methods under the theoretical foundations of health behavior informatics.

This direction of the CH development needs studies to investigate what kind of contracting strategies and incentives could facilitate implementations of CH tools that enable cost containment by keeping people longer out of health care facilities or providers. The integration of CH services with new promising cost containment and quality improvement policies should be a research priority, and new business models should be designed. Field studies should be promoted to collect evidence and understand needs. Health economics and finance should be revised based on new political guidance.

CH technologies can be employed for adapting public health policies, addressing a broader health-related impact, which also involves transitions to new models of care. The availability of CH data combined with *big data analytics* can be of added value toward supporting the learning health system cycle [41].

Besides the cost and business perspective, it is essential to recognize the role and the rising needs for CH education, entailing for interprofessional aspects. CH education is related to preparing the stakeholders and addressing barriers and concerns as well as contextual factors. Elaboration of new curricula for HCPs and health researchers, while addressing CH literacy for citizens in an organized and inspiring manner, could have a transformative power toward CH impact.

### Limitations

A limitation of this study is the lack of extensive evaluation of the proposed CHIF framework. In addition, the lack of standard terminology may pose challenges toward extended use of the framework for comparison and new knowledge elicitation. The addition of formal descriptions and semantics and the link to standard terminologies is considered a necessary next step. The adoption of standards and semantics is expected to alleviate some of the possible difficulties and ambiguities related to the current implementation and lead to broader use and evaluation of the framework.

### Conclusions

CH technologies offer new vehicles for implementing *anytime and anywhere* health and care services. Being an emerging and diverse field, CH will benefit from the disambiguation of concepts. In addition, scaling up of these services is closely related to a means for understanding and measuring their impact. In this regard, this study introduces CHIF, a framework for CH impact assessment that contributes to the formalization of the CH domain, also paving the way toward the introduction of methods for measuring and comparison in multiple scales and dimensions related to CH outcomes. CHIF can evolve toward the creation of a CH impact tool and contribute to the generation of a service registry for further comparison and investigation.

## Appendix

Multimedia Appendix 1 Workshops description.

Multimedia Appendix 2 Screen captures of the electronic questionnaire based on the connected health impact framework (CHIF).

Multimedia Appendix 3 The electronic questionnaire.

Multimedia Appendix 4 The electronic questionnaire summarized answers.

## Abbreviations

CH: connected health
CHIF: connected health impact framework
COST: Cooperation in Science and Technology
eHealth: electronic health
ENJECT: European Network for the Joint Evaluation of Connected Health Technologies
HCP: health care professional
HIA: health impact assessment
HL7: health level 7
MAST: Model for Assessment of Telemedicine
mHealth: mobile health
