# Supplementary material for: Connected Health Services: Framework for an Impact Assessment
Source: J Med Internet Res. 2019 Sep 3;21(9):e14005. doi: 10.2196/14005 (PMC6751095; doi:10.2196/14005)
Supplement: Multimedia Appendix 1 [file jmir_v21i9e14005_app1.docx]

# Supplementary Material for ‘CHIF: A CONNECTED HEALTH IMPACT FRAMEWORK’

## **Appendix 1. Workshops description**

This appendix provides a description of the 2 WG1 Workshops oriented to CH impact that took place within ENJECT COST Action

**Workshop 1 details:** 18 ENJECT representatives participated, including PhD students, academia and industry professionals, from medicine, physiotherapy, business, and ICT backgrounds. The workshop lasted for a whole day. It was a three-phase procedure. In phase 1, participants had to individually reflect on a series of questions (listed in Appendix 1) and answer in written form. The questions were organized In 3 groups. In phase 2, the participants were split into 3 groups and each group discusses the answers that were provided by the group member so as reach a fusion of ideas, a consensus or a ranking/prioritization within group. In phase 3, a plenary discussion took place. Each group’s rapporteur presented their results to the whole workshop participants. A written report was also provided.

The questions that have been employed in the first workshop are presented below. The employed questions have been organized into 4 themes, namely *Impact Description*, *Means & mechanisms for achieving CH impact*, *Evaluation*, and *Impact & models of care*. The questions and the theme they belong to are listed in the table that follows.

| **Impact description** | Foreseen CH outcomes that can lead to health impact related to? (e.g., prevention of.., risk of safety, change….) |
| --- | --- |
|  | Is it just the impact of consumer electronics in daily life? Or? |
|  | Cases where there can be impact specific to CH and not previously achieved-addressed by ehealth/mhealth |
| **Means & mechanisms for achieving CH impact** | In what way does CH target and opens the opportunity for health impact (e.g., by means of new services, …) |
|  | To what extent is CH deployed, and its impact achieved so far (e.g pilot) |
|  | How much are the stakeholders prepared? (citizens? carers? the state?) |
|  | What are the stakeholders’ concerns – more than before? (legal? accountability?) |
|  | What are the barriers to achieving impact? |
|  | What future research activities can facilitate CH impact? |
| **Evaluation** | How can CH impact be evaluated? (outcomes? indicators? metrics?) |
|  | What are the contextual factors influencing its adoption (geographical? literacy? ) |
|  | What is the link with new models of care (integrated care?) |
| **Impact & models of care** | Is there a connection with pathway modeling and impact assessment? |

**Workshop 2 details**: 10 ENJECT representatives participated, including PhD students, academia and industry professionals, from multiple disciplines, not coinciding with the participants of the first workshop. The workshop lasted ½ day.
