# Supplementary material for: Connected Health Services: Framework for an Impact Assessment
Source: J Med Internet Res. 2019 Sep 3;21(9):e14005. doi: 10.2196/14005 (PMC6751095; doi:10.2196/14005)
Supplement: Multimedia Appendix 2 [file jmir_v21i9e14005_app2.docx]

# Supplementary Material for ‘CHIF: A CONNECTED HEALTH IMPACT FRAMEWORK’

## **Appendix 2. Screen captures of the electronic questionnaire based on CHIF framework**


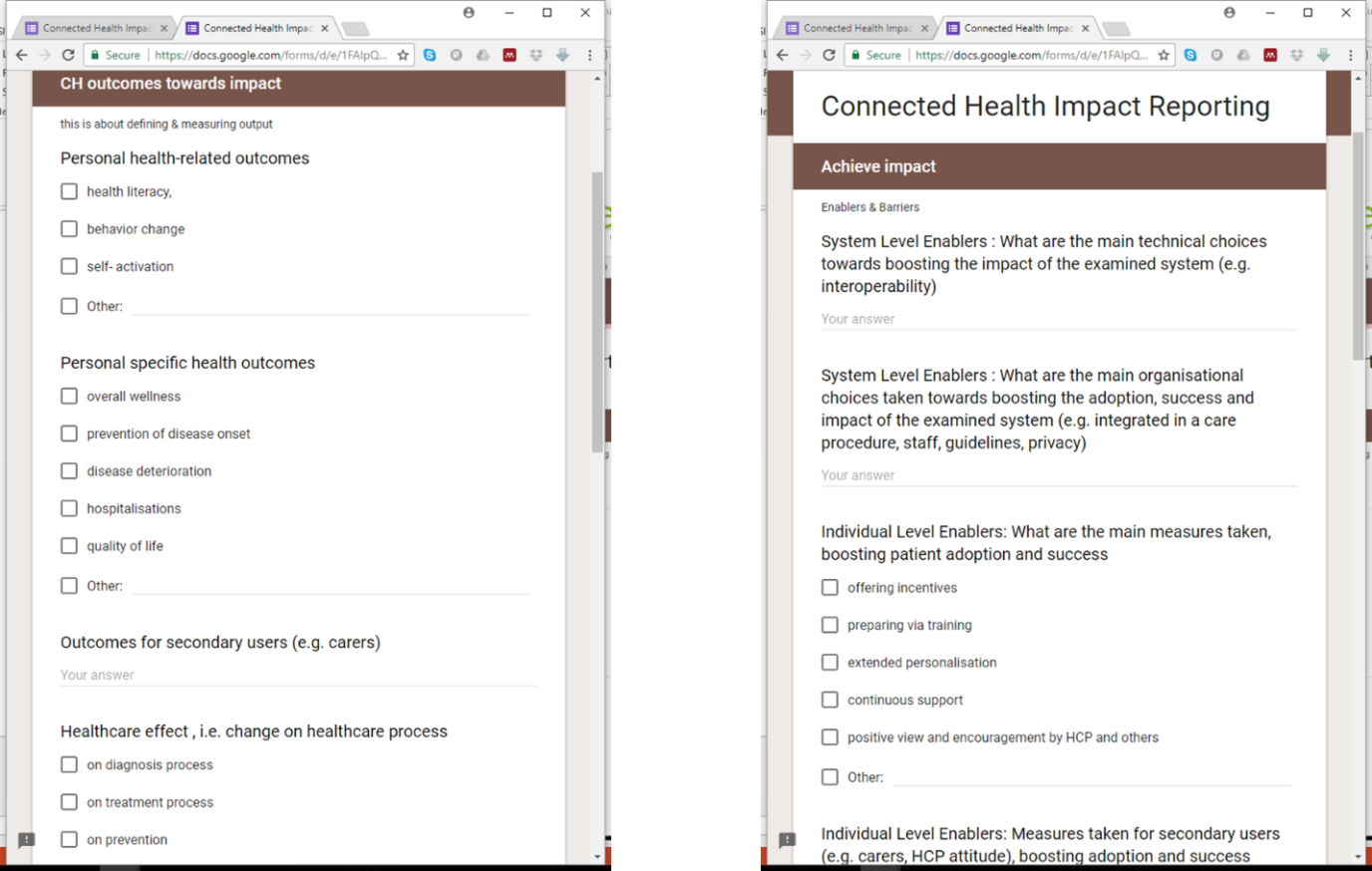


The full questionnaire can be found at <https://goo.gl/forms/Ovi6iXJ26sOFZPiS2>.
