# Supplementary material for: Connected Health Services: Framework for an Impact Assessment
Source: J Med Internet Res. 2019 Sep 3;21(9):e14005. doi: 10.2196/14005 (PMC6751095; doi:10.2196/14005)
Supplement: Multimedia Appendix 3 [file jmir_v21i9e14005_app3.pdf]

Connected Health Impact Reporting

Background
Connected Health (CH) as a new paradigm looks after the individual and community health in a "connected" and "holistic" manner by leveraging a variety of technologies to do so.
CH has been seen as a promising vehicle for integrating telehealth and integrated care services, in other words the health-related services that cover all conditions from the healthy subject as a health service consumer, to the chronic patient as an integrated care and telecare service beneficiary.
This reorganization of services around the person-citizen has been expected to bring high impact in the health domain. A series of concerns within the Connected Health domain need to be better addressed, in order to better reach stakeholders: contextual factors influencing the impact of care models, the cost savings associated with connected health solutions, the sustainability of a connected health ecosystem. Overall, there is need to shed some light regarding the concepts of CH impact, and ENJECT WG1 is working in this direction.

Aim
This questionnaire aims to explore how CH impact related drivers and outcomes can be reported. You are asked to fill in information regarding a CH technology or service you are aware of (reporting of existing one), or one that you are designing/thinking of (reporting of a future one) , with respect to its impact.

* Required

Describe CH system

describe a system that exists and you know about, or one that you are thinking about

1. **Application description/aim** *

____
____
____
____
____

2. **The main function(s) of this system**

Check all that apply.

- [ ] monitoring
- [ ] training and knowledge building
- [ ] disease management
- [ ] lifestyle management
- [ ] Other: _______________________________

3. **The main process(es) of this system**

Check all that apply.

- [ ] receive patient reports
- [ ] receive patient measurements
- [ ] give automated feedback
- [ ] give Healthcare Professional feedback
- [ ] involve social interaction
- [ ] Other: _______________________________

4. **The primary goal - what will the user achieve**

____

5. **Control / Governance**

Mark only one oval.

- ◯ personal (citizen/consumer) service,
- ◯ health care service
- ◯ social service
- ◯ Other: ____________________________

6. **Level of evidence**

Mark only one oval.

- ◯ under design
- ◯ technical validation
- ◯ clinical testing

CH system user profiles

Who is this meant for?

7. **Life Period**

Check all that apply.

- ☐ Younger
- ☐ Working
- ☐ Retired
- ☐ In Dependency
- ☐ Other: ____________________________

8. **Gender**

Mark only one oval.

- ◯ Female
- ◯ Male
- ◯ All

9. **Health status**

Check all that apply.

- ☐ Healthy
- ☐ Chronic
- ☐ Comorbid
- ☐ Acute
- ☐ Other: ____________________________

10. **Life Context (e.g. suitable for people in certain areas, at home/work, under certain socioeconomic conditions, etc)**

11. **Other, secondary users**
    Check all that apply.

- [ ] carer
- [ ] healthcare professional
- [ ] researcher
- [ ] health policy
- [ ] industry
- [ ] Other:

CH outcomes towards impact

this is about defining & measuring output - what is expected to change with this service

12. **Personal health-related outcomes**
    Check all that apply.

- [ ] health literacy
- [ ] behavior change
- [ ] self- activation
- [ ] Other:

13. **Personal specific health outcomes**
    Check all that apply.

- [ ] overall wellness
- [ ] prevention of disease onset
- [ ] disease deterioration
- [ ] hospitalisations
- [ ] quality of life
- [ ] Other:

14. **Outcomes for secondary users (e.g. carers)**

15. **Healthcare effect , i.e. change on healthcare process**
Check all that apply.

- [ ] on diagnosis process
- [ ] on treatment process
- [ ] on prevention
- [ ] on stratification of patients
- [ ] Other: ____________________

16. **Effect on human interaction & relations**
Check all that apply.

- [ ] patient-doctor communication & shared decisions
- [ ] patients / citizens information and experience sharing
- [ ] patient / carer dependency
- [ ] Other: ____________________

17. **Effect on new medical knowledge about health and disease**

____
____
____
____
____

18. **Horizontal & socioeconomic effect**
Check all that apply.

- [ ] cost of care
- [ ] access to services
- [ ] public health policy
- [ ] Other: ____________________

19. **Related industrial activity & business growth**

____
____
____
____
____

20. **Can you specify in what time scales the different abovementioned effects may occur**

____
____
____
____
____

21. **Do you have other suggestions about reporting the CH outcomes and impact**

Achieve impact

Enablers & Barriers

22. **System Level Enablers : What are the main technical choices towards boosting the impact of the examined system (e.g. interoperability)**

23. **System Level Enablers : What are the main organisational choices taken towards boosting the adoption, success and impact of the examined system (e.g. integrated in a care procedure, staff, guidelines, privacy)**

24. **Individual Level Enablers: What are the main measures taken, boosting patient adoption and success**

Check all that apply.

- [ ] offering incentives
- [ ] preparing via training
- [ ] extended personalisation
- [ ] continuous support
- [ ] positive view and encouragement by HCP and others
- [ ] Other: ____________________

25. **Individual Level Enablers: Measures taken for secondary users (e.g. carers, HCP attitude), boosting adoption and success**

26. **System Level Barriers: what are the technical problems and barriers, preventing system adoption and success**
Check all that apply.

- [ ] usability, issues, and concerns
- [ ] data security
- [ ] information workflow and integration
- [ ] Other: ____________________

27. **System Level Barriers: what are the organisational problems and barriers, preventing system adoption and success**
Check all that apply.

- [ ] reimbursement system
- [ ] incompatibility with health care system
- [ ] staff related issues
- [ ] legal , privacy, liability
- [ ] Other: ____________________

28. **Individual Level Barriers for secondary users (e.g. carers, HCP) preventing adoption**

____
____
____
____
____

Regarding the value proposition addressed below, see also the following link

http://www.mckinsey.com/industries/pharmaceuticals-and-medical-products/our-insights/capturing-value-from-connected-health

29. **The systems value proposition. Desirable for the patient because:**

____
____
____
____
____

30. **The systems value proposition. Desirable for the secondary user (HCP, carer...) because:**

____
____
____
____
____

31. **The systems value proposition. Technically feasible because :**

32. **The systems value proposition. Organizationally feasible because :**

33. **The systems value proposition. Viable because :**

Other comments

34. **Do you have other suggestions about reporting measures to achieve impact**

Contact

Contact Enject for any related question : enject@ucd.ie

35. **If you feel comfortable with this, and you are interested in WG1 work about CH impact, leave your name and email address with us.**
