# Supplementary material for: Connected Health Services: Framework for an Impact Assessment
Source: J Med Internet Res. 2019 Sep 3;21(9):e14005. doi: 10.2196/14005 (PMC6751095; doi:10.2196/14005)
Supplement: Multimedia Appendix 4 [file jmir_v21i9e14005_app4.pdf]

# Connected Health Impact Reporting

5 responses

## Describe CH system

### Application description/aim

5 responses

To send health information

personal trainer

Collecting health records and drug intake for elderly people

The system is called SPLENDID and it is an mHealth intervention for obesity and eating disorders prevention through eating and physical activity modification.

The end-user interfaces a smartphone application which allow self-reporting and sensory acquisition of eating behavior and physical activity data. The data are sent to a server where the behavioral patterns of the end-user is analyzed automatically and presented to a healthcare professional.

Through goal setting, feedback and motivational messages the potentially obesogenic behavior of the end-user is regulated/modified towards more healthy eating and physical activity habits/patterns.

to support exercise rehabilitation at home

### The main function(s) of this system

5 responses

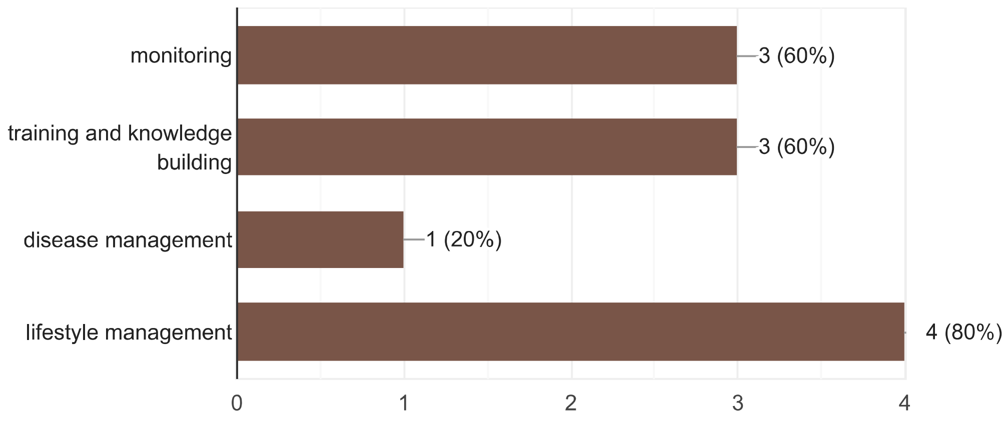

## The main process(es) of this system

5 responses

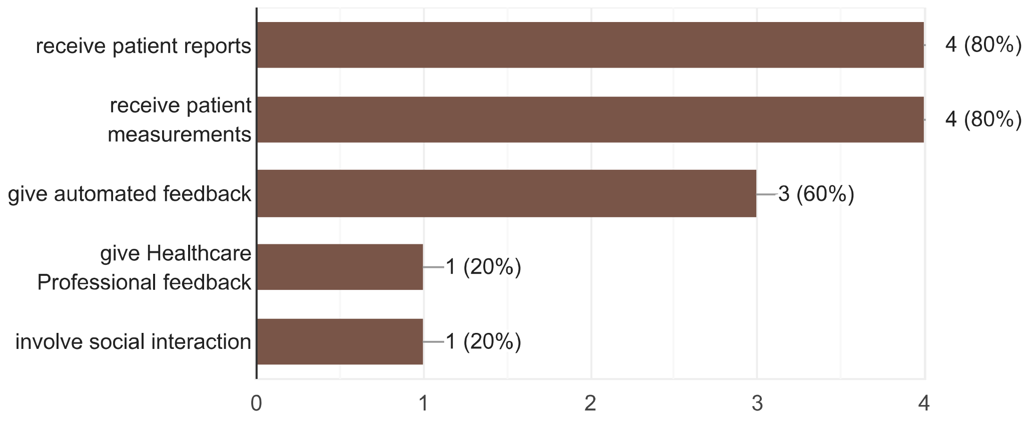


## The primary goal - what will the user achieve

3 responses

Send health information

Long-term modification of his/her harmful eating and physical activity behavior

adherence to recommended exercise

## Control / Governance

5 responses

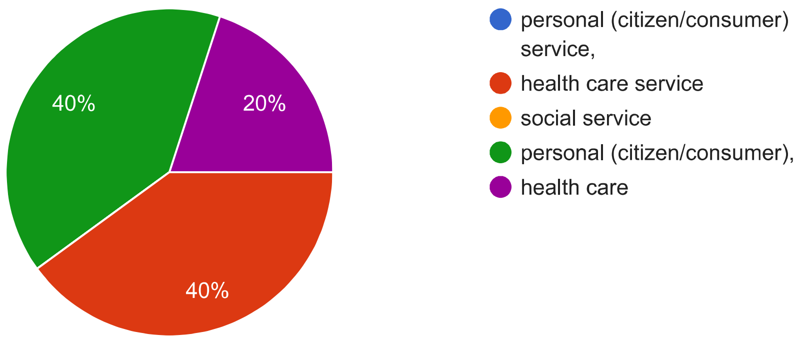

## Level of evidence

5 responses

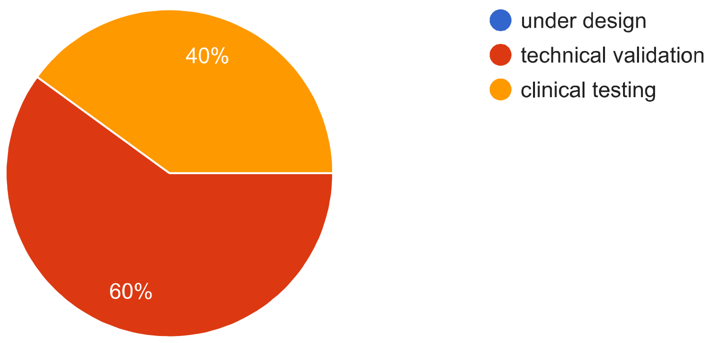


# CH system user profiles

## Life Period

5 responses

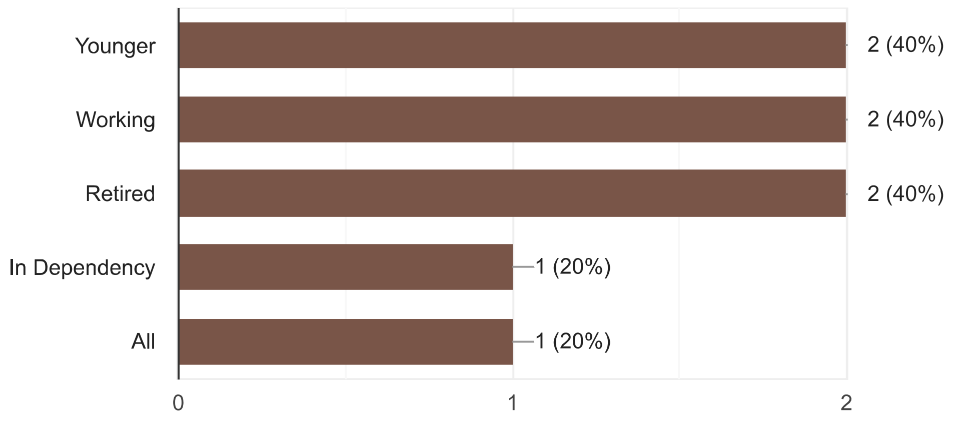


## Gender

5 responses

## Health status

5 responses

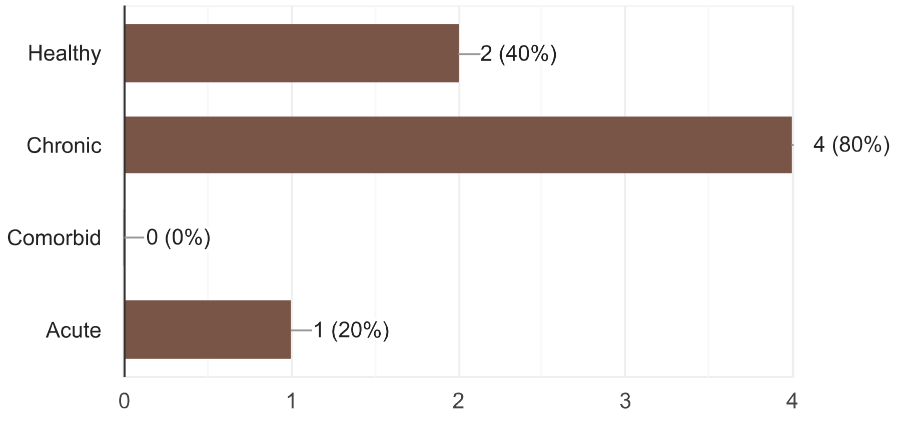


## Life Context (e.g. suitable for people in certain areas, at home/work, under certain socioeconomic conditions, etc)

4 responses

All

no particular

suitable for people in certain areas

mostly remote areas

## Other, secondary users

5 responses

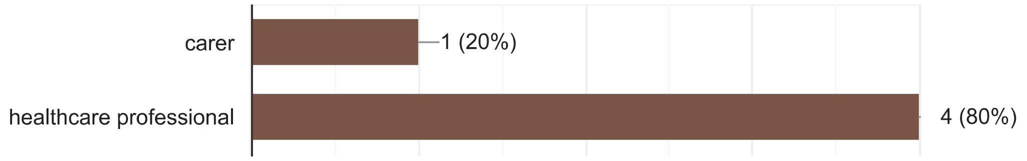


CH outcomes towards impact

## Personal health-related outcomes

5 responses

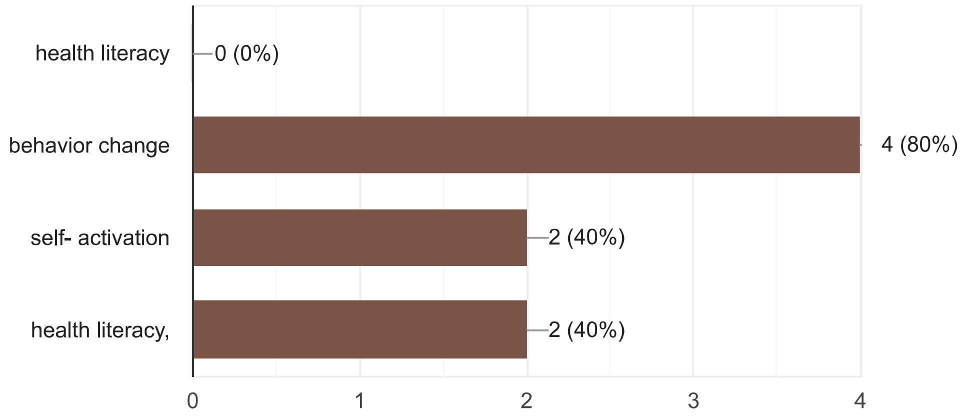


## Personal specific health outcomes

5 responses

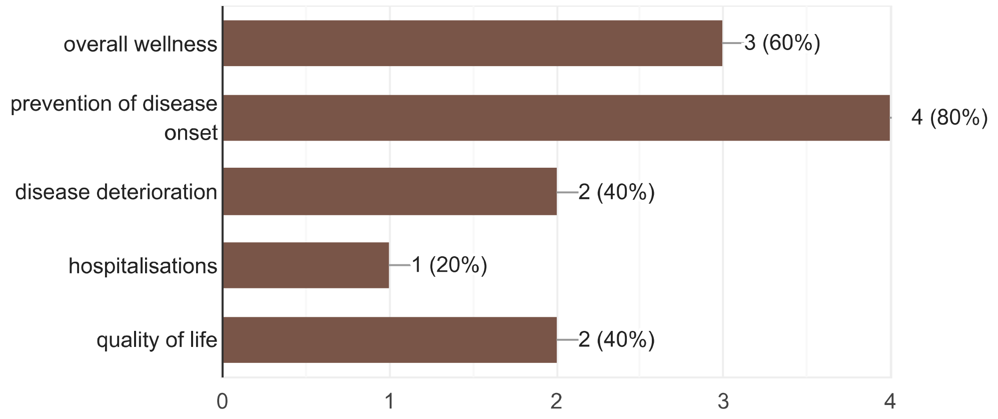


## Outcomes for secondary users (e.g. carers)

2 responses

knowledge for policy makers

More data available for analysis of patterns

## Healthcare effect , i.e. change on healthcare process

5 responses

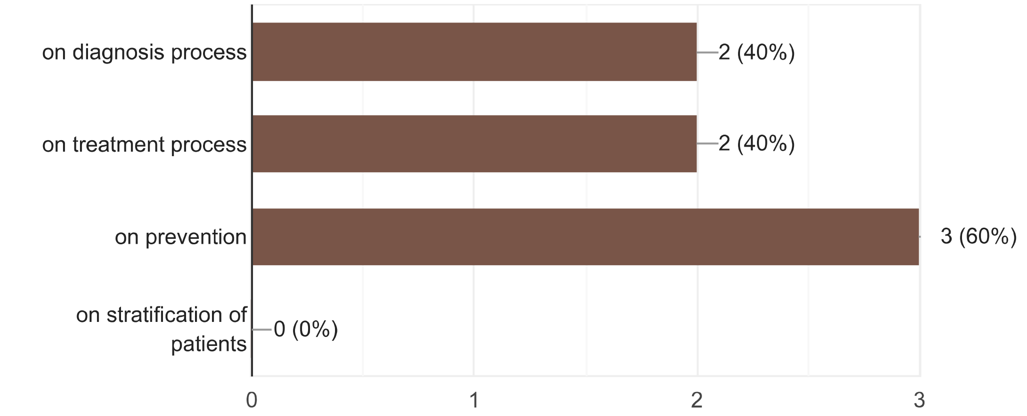


## Effect on human interaction & relations

4 responses

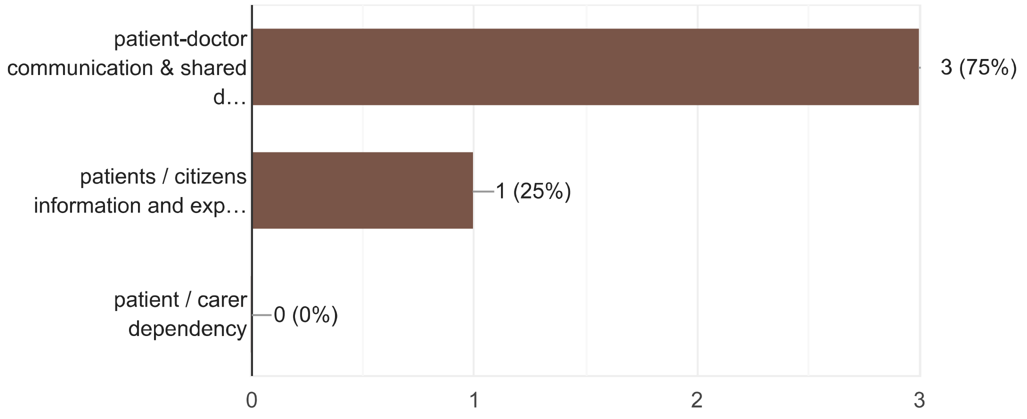


## Effect on new medical knowledge about health and disease

2 responses

more data available

knowledge on exercise adherence and factors affecting it

## Horizontal & socioeconomic effect

5 responses

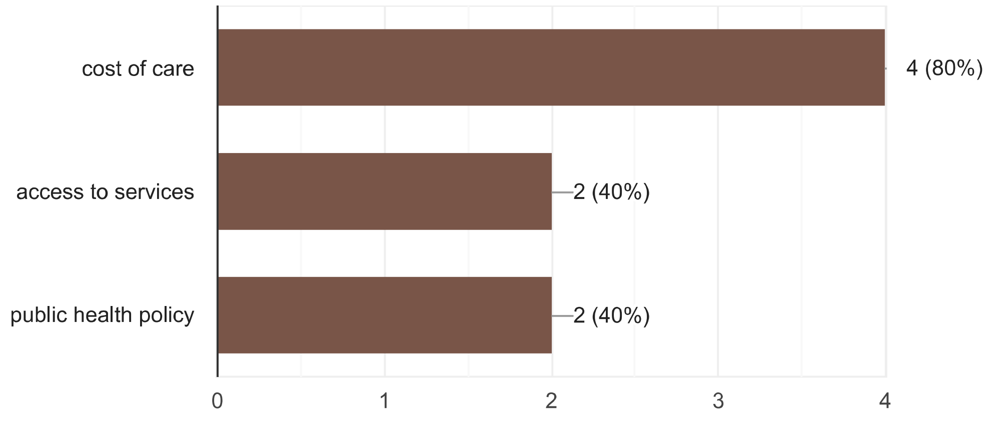


## Related industrial activity & business growth

1 response

sensors and mhealth

## Can you specify in what time scales the different abovementioned effects may occur

1 response

from months to years probably

Do you have other suggestions about reporting the CH outcomes and impact

0 responses

No responses yet for this question.

## Achieve impact

System Level Enablers : What are the main technical choices towards boosting the impact of the examined system (e.g. interoperability)

2 responses

cloud based anonymous data sharing

not complicated use

System Level Enablers : What are the main organisational choices taken towards boosting the adoption, success and impact of the examined system (e.g. integrated in a care procedure, staff, guidelines, privacy)

1 response

low price or prescribed service

Individual Level Enablers: What are the main measures taken, boosting patient adoption and success

4 responses

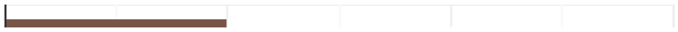

## Individual Level Enablers: Measures taken for secondary users (e.g. carers, HCP attitude), boosting adoption and success

0 responses

No responses yet for this question.

## System Level Barriers: what are the technical problems and barriers, preventing system adoption and success

4 responses

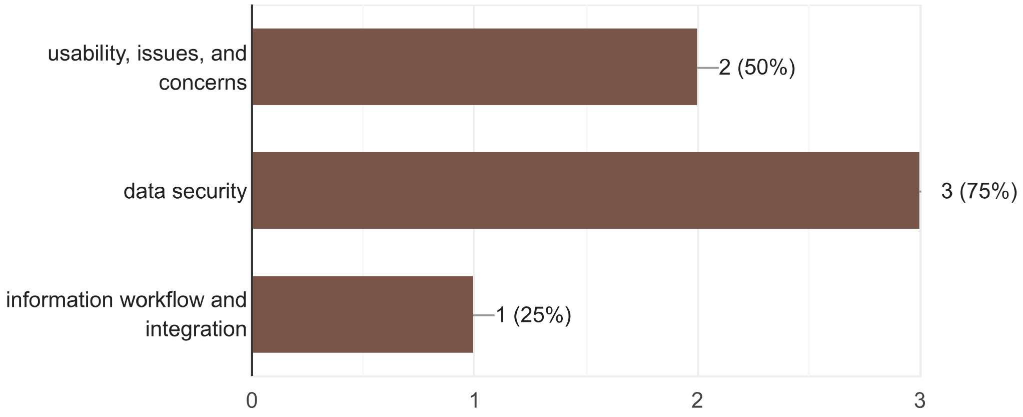


## System Level Barriers: what are the organisational problems and barriers, preventing system adoption and success

4 responses

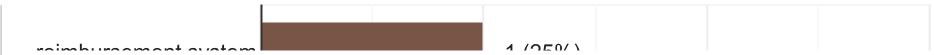

Individual Level Barriers for secondary users (e.g. carers, HCP) preventing adoption

0 responses

No responses yet for this question.

Regarding the value proposition addressed below, see also the following link

The systems value proposition. Desirable for the patient because:

0 responses

No responses yet for this question.

The systems value proposition. Desirable for the secondary user (HCP, carer...) because:

0 responses

No responses yet for this question.

The systems value proposition. Technically feasible because :

0 responses

No responses yet for this question.

The systems value proposition. Organizationally feasible because :

0 responses

No responses yet for this question.

## The systems value proposition. Viable because :

0 responses

No responses yet for this question.

### Other comments

## Do you have other suggestions about reporting measures to achieve impact

0 responses

No responses yet for this question.

### Contact

## If you feel comfortable with this, and you are interested in WG1 work about CH impact, leave your name and email address with us.

0 responses

No responses yet for this question.

This content is neither created nor endorsed by Google. Report Abuse - Terms of Service

Google Forms
